# A randomised controlled trial to evaluate and optimize the use of antiplatelet agents in the perioperative management in patients undergoing general and abdominal surgery- the APAP trial (ISRCTN45810007)

**DOI:** 10.1186/1471-2482-11-7

**Published:** 2011-03-03

**Authors:** Dalibor Antolovic, Christoph Reissfelder, Anastasia Rakow, Pietro Contin, Nuh N Rahbari, Markus W Büchler, Jürgen Weitz, Moritz Koch

**Affiliations:** 1 From the Department of Surgery, University of Heidelberg, Germany

## Abstract

**Background:**

Due to the increase of cardiovascular diseases acetylsalicylic acid (ASA) has become one of the most frequently prescribed drugs these days. Despite the rising number of patients with ASA medication presenting for elective general and abdominal surgery and the potentially increased risk of hemorrhage in these patients, there are no clear, evidence-based guidelines for the perioperative use of antiplatelet agents. The present randomised controlled trial was designed to evaluate the safety and optimize the use of ASA in the perioperative management of patients undergoing general and abdominal surgery.

**Methods/Design:**

This is a two-arm, monocenter randomised controlled trial. Patients scheduled for elective surgical treatment (i.e. inguinal hernia repair, cholecystectomy and colorectal resections) with ASA as a permanent medication are randomised equally to perioperative continuation or discontinuation of ASA. Patients who are randomised in the discontinuation group stop the administration of ASA five days prior to surgical treatment and start intake of ASA on postoperative day 5. Fifty-two patients will be enrolled in this trial. The primary outcome is the incidence of postoperative bleeding and cardiovascular events at 30 days after surgery. In addition a set of general as well as surgical variables are analysed.

**Discussion:**

This is a randomised controlled two-group parallel trial designed to assess the safety and optimize the use of ASA in the perioperative management of patients undergoing general and abdominal surgery. The results of this pilot study build the basis for a confirmative randomised controlled trial that may help to clarify the use and potential risk/benefits of perioperative ASA medication in patients undergoing elective surgery.

**Trial registration:**

The trial is registered with Current Controlled Trials ISRCTN45810007.

## Background

The individual risk for perioperative bleeding in abdominal and general surgery is influenced by multiple factors. Such factors include extent and type of surgery as well as patient-related features such as age, co-morbidities and perioperative medication. Considering the current demographic development surgeons are being confronted with older patients presenting with multiple co-morbidities and medications. Antiplatelet agents and in particular acetylsalicylic acid (ASA) were shown to be beneficial in a variety of atherothrombotic diseases [1-4] and are therefore among the most frequently prescribed drugs these days. However, due to the increased risk of hemorrhage the perioperative use of ASA has remained an issue of ongoing discussion [5-13].

Randomised controlled trials (RCT) that allow evidence-based recommendations regarding the perioperative use and risk of ASA in general and visceral surgery are still lacking. Therefore, the decision to balance possible thromboembolic risks after withdrawal of ASA versus bleeding risks after continuation of ASA during the perioperative period is rather based on the individual experience and personal opinion of the treating physician and/or surgeon.

Apart from severe bleeding episodes complicating the clinical course after an operation, thromboembolic events also account for serious consequences for the individual patient. Presumably, the routinely performed discontinuation of the regular ASA medication in the perioperative setting entails to an increased incidence of thromboembolic events. Retrospective analyses have shown that acute cardiovascular events occur in 2-10% of patients after perioperative discontinuation of ASA, mostly within the first eight days after stopping the long-term anticoagulation regimen.

This is a randomised controlled two-group parallel trial designed to assess the safety and optimize the use of ASA in the perioperative management of patients undergoing general and abdominal surgery. The results of this trial will be used to perform a valid power calculation and to design a confirmative RCT with a higher number of enrolled patients. This may help to clarify the use and potential risk/benefits of perioperative ASA medication in patients undergoing elective surgery.

## Methods and Design

This study has been designed as a prospective randomised controlled, single centre trial with two study arms.

After the study has been approved by the ethics committee of the University of Heidelberg and registration of the protocol at an international registry (ISRCTN45810007) patients on continuous medication with ASA undergoing elective general or abdominal surgery at the Department of General, Visceral and Transplant Surgery, University of Heidelberg, Germany are being screened for participation in this study. Randomisation is conducted using opaque, serially numbered envelopes. The envelopes are being supplied by the Clinical Trial Centre of the Department of Surgery, University of Heidelberg, Germany.

### Participants and Trial population

This study includes patients over 18 years of age who require long-term ASA therapy and are scheduled for the following procedures:

1.) Inguinal Hernia Repair (e.g. Shouldice repair, Lichtenstein's repair, laparoscopic hernia repair)

2.) Cholecystectomy (open and laparoscopic approach)

3.) Elective Colorectal Surgery (right or left hemicolectomy, sigmoid resection, low anterior resection)

Written informed consent is obtained prior to inclusion into the trial.

Patients with ASA medication who were advised to continue antiplatelet therapy throughout the perioperaive period due to medical indication (e.g. shortly after coronary stent implantation, bleeding disorders, etc.) or who were cardiologically evaluated as being a high-risk patient were excluded from this trial. Moreover, emergency procedures, simultaneous participation in another clinical trial, withdrawn or missing written consent and/or a mental condition rendering the subject incapable of understanding the nature, scope, and consequences of the trial lead to exclusion from this study. The eligibility criteria are:

### Inclusion Criteria

- Age equal or greater than 18 years

-Patient has given written informed consent

-Patient is scheduled for elective surgery based on an indication for either inguinal hernia repair, cholecystectomy or colorectal surgery procedures

-Patient treated with ASA on a long-term-medication basis

-Low- or intermediate-risk patient according to cardiological evaluation

### Exclusion Criteria

-High-risk patient according to cardiological evaluation (e.g. necessary perioperative continuation of the ASA application shortly after coronary stent implantation, interfering bleeding disorders etc.)

-Emergency procedure

-Withdrawn or missing written consent

-Simultaneous participation in another clinical trial with interference of intervention and outcome

-Severe psychiatric or neurologic diseases or a mental condition rendering the patient incapable of understanding the nature, scope, and consequences of the trial.

-Concomitant treatment with coumarin-type-anticoagulation or clopidogrel

### Trial interventions

Patients are randomized into one the following two study arms:

Arm 1: Continuation of ASA during the perioperative period

Arm 2: Discontinuation of ASA five days prior to surgery and resumption of ASA intake on postoperative day 5.

### Primary outcomes/secondary outcomes (Table 1)

**Table 1:** Primary and secondary outcomes

**Primary endpoints**	**Definition and assessment of outcomes**
Perioperative thromboembolic event	
Perioperative bleeding complication	*Occurrence of critical blood-loss requiring surgical intervention or the application of coagulation products (FFP, coagulation factors) to normalize the INR*
**Secondary endpoints**	**Definition and assessment of outcomes**
Operation time [min]:	Time from skin incision to placement of last skin staple/suture.
Intraoperative blood loss [ml]:	Blood loss observed from skin incision to placement of last staple/suture.
Duration of postoperative hospital stay [days]:	Time from day of surgery to day of discharge.
Morbidity/	
Surgical complications	
Medical complications	
Peri-/postoperative need for blood/coagulation products	
Re-laparotomy/laparoscopy	Re-operation within the time of index hospitalization respectively within the time of follow-up after the index operation.
In-hospital mortality:	Death due to any reason within the patient's initial hospital stay.
Readmisson	

The primary objective of the present randomised controlled trial is to evaluate the safety of continuous use of ASA during the perioperative period of patients undergoing elective general and abdomina surgery with the ultimate aim to generate high-quality data for the development of evidence-based guidelines on the perioperative use of ASA.

The primary outcome is the incidence of perioperative bleeding episodes and clinically apparent thromboembolic events within 30 days after surgery. Secondary endpoints are the duration of the surgical intervention, intraoperative blood loss, transfusion requirements, length of hospital stay (d), medical and surgical morbidity, and the necessity of readmission.

#### Sample size

Owing to the lack of data that would allow a valid sample size calculation, the present trial was designed as a randomised controlled pilot trial. A total of fifty-two consecutive patients with continous low-dose medication of ASA, who are scheduled for elective general and abdominal surgery, are enrolled in the present study and randomly allocated to either study arm. This pilot trial generates data for the power calculation of a subsequent RCT with a confirmative design. For the pilot phase of this trial the number of patients to be enrolled was arbitrary defined by the investigators.

#### Statistical analysis

The statistical analysis will be performed on the basis of baseline data such as age, gender, type of surgery, complications/adverse effects and duration of hospital stay. There will be a report of the mean and standard deviation for variables with continuous measures, whereas we will report numbers and percent for categorical data. Statistical computations will be performed with JMP (SAS Institute, Cary, NC) and SPSS (SPSS Inc., Chicago, IL).

#### Ethical approval and registration

The ethics committee of the University of Heidelberg approved this clinical study (Reference number S003-2008). The trial is registered at an international study registry http://www.controlled-trials.com ISRCTN45810007).

## Competing interests

The authors declare that they have no competing interests with this study.

## Authors' contributions

DA, CR, AR and MK designed the study and wrote the original protocol, PC and NNR help in enrolling patients in this study and MWB and JW are scientifically responsible for the study. All authors read and approved the final manuscript.

## Pre-publication history

The pre-publication history for this paper can be accessed here:

http://www.biomedcentral.com/1471-2482/11/7/prepub
